# Altitude-induced effects on neuromuscular, metabolic and perceptual responses before, during and after a high-intensity resistance training session

**DOI:** 10.1007/s00421-023-05195-3

**Published:** 2023-05-20

**Authors:** Gonzalo Márquez, David Colomer, Cristina Benavente, Luis Morenilla, Carlos Alix-Fages, Paulino Padial, Belén Feriche

**Affiliations:** 1 Department of Physical Education and Sport, Faculty of Sports Sciences and Physical Education, University of A Coruna, Avda. Ernesto Che Guevara, 121-Pazos-Liáns, 15179 Oleiros, A Coruña Spain; 2 Department of Physical Education and Sport, Faculty of Sports Sciences, University of Granada, Granada, Spain; 3 Applied Biomechanics and Sports Technology Research Group, Autonomous University of Madrid, Madrid, Spain

**Keywords:** Hypoxia, Transcranial magnetic stimulation, Strength training, Corticospinal excitability, Intracortical inhibition

## Abstract

**Purpose:**

We tested if an acute ascending to 2320 m above sea level (asl) affects corticospinal excitability (CSE) and intracortical inhibition (SICI) measured with transcranial magnetic stimulation (TMS) at rest, before, during and after a traditional hypertrophy-oriented resistance training (*R*_T_) session. We also explored whether blood lactate concentration (BLa), ratings of perceived exertion (RPE), perceived muscular pain and total training volume differed when the *R*_T_ session was performed at hypoxia (H) or normoxia (N).

**Methods:**

Twelve resistance-trained men performed eight sets of 10 repetitions at 70% of one repetition maximum of a bar biceps curl at N (SpO_2_ = 98.0 ± 0.9%) and H (at 2320 asl, SpO_2_ = 94.0 ± 1.9%) in random order. Before each session, a subjective well-being questionnaire, the resting motor threshold (rMT) and a single pulse recruitment curve were measured. Before, during and after the *R*_T_ session, BLa, RPE, muscle pain, CSE and SICI were measured.

**Results:**

Before the *R*_T_ session only the rMT differed between H (− 5.3%) and N (ES = 0.38). RPE, muscle pain and BLa increased through the *R*_T_ session and were greater at H than N (12%, 54% and 15%, respectively) despite a similar training volume (1618 ± 468 kg vs. 1638 ± 509 kg). CSE was reduced during the *R*_T_ session (~ 27%) but recovered ten minutes after, regardless of the environmental condition. SICI did not change after any *R*_T_ session.

**Conclusions:**

The data suggest that acute exposure to moderate hypoxia slightly increased the excitability of the most excitable structures of the corticospinal tract but did not influence intracortical or corticospinal responses to a single *R*_T_ session.

## Introduction

Altitude training is a strategy commonly used by high-level athletes to improve their performance in endurance activities such as running, swimming, or cycling. However, altitude or hypoxic exposure has also gained popularity as a promising strategy to maximize neuromuscular performance (Tomazin et al. 2021). In this regard, research has shown improvements in neuromuscular adaptations (e.g.: maximal strength, power and muscle hypertrophy) after resistance training (*R*_T_) programs performed under hypoxic conditions (Feriche et al. 2017; Ramos-Campo et al. 2018). For example, an acute ascent to 2320 m above sea level (asl) induced positive effects on drop jump performance after 6 h of exposure (Štirn et al. 2022). Furthermore, results from Tomazin et al. (2021) demonstrated that 3 weeks of power-oriented strength training (loaded countermovement jumps − 30 to 40% of the one repetition maximum [1RM]–, back squat and dead lift against 70–90% of the RM) performed at moderate altitude (i.e.: 2320 m asl) produced a more intense and positive effect on contractile muscle properties (e.g.: twitch contraction time) than similar training performed at sea level.

The beneficial effects of *R*_T_ under hypoxia are thought to be mediated by increases in metabolic stress (Scott et al. 2016b), which is likely related to higher motor unit recruitment (Scott et al. 2017). During the performance of maximal and submaximal contractions in acute hypoxia, there is a larger anaerobic metabolism contribution to ATP synthesis due to the decreased oxygen availability, which leads to a greater metabolite build-up (Balsom et al. 1994; Kon et al. 2010; Feriche et al. 2017). This hypoxia-induced intramuscular metabolic disturbance evokes a rise in discharge frequency of group III/IV muscle afferents (Rotto and Kaufman 1988; Scott et al. 2014) that increases their inhibitory influence over central motor drive (Amann and Dempsey 2008), accelerating the decline in voluntary force output. To compensate for group III/IV inhibition and premature fatigue over firstly recruited motor units due to the metabolic acidosis, a greater supraspinal drive is needed to recruit additional motor units and maintain force production (Taylor et al. 2016). Therefore, not only the increased motor unit recruitment acts as a driving stimulus for a greater hypertrophy response (Feriche et al. 2017), but also the increased supraspinal drive could act as a potential contributing factor for greater neuromuscular adaptations.

Previous studies have used transcranial magnetic stimulation (TMS) to measure corticospinal excitability (CSE) or intracortical inhibition changes in response to a single *R*_T_ session (Mason et al. 2020; Colomer‐Poveda et al. 2020). These acute changes have been suggested to be related to the strengthen of cortical and corticospinal connections to the trained muscles due to use-dependent plasticity, which may act as the early neural adaptations to *R*_T_ (Nuzzo et al. 2016; Colomer-Poveda et al. 2019). *R*_T_ variables such as intensity (Mason et al. 2020; Colomer‐Poveda et al. 2020), volume (Colomer-Poveda et al. 2019) or muscle contraction type (Latella et al. 2019) may influence those acute changes to a single *R*_T_ session. For example, it has been previously shown that greater training intensities, such as 75% of maximum voluntary contraction (MVC) compared to 50% or 25% of MVC, lead to greater increases in CSE after an acute *R*_T_ session (Colomer-Poveda et al. 2019; Mason et al. 2020; Colomer‐Poveda et al. 2020). Moreover, those *R*_T_ protocols that used higher intensities (75% of 1RM) have shown superior neuromuscular adaptations after 4 weeks of training compared to those *R*_T_ programs that used lower intensities (i.e.: 25% of 1RM), even when total volume was matched (Colomer‐Poveda et al. 2021). Therefore, other factors that may increase supraspinal drive during *R*_T,_ such as hypoxia, may increase the acute changes in the corticospinal pathway compared to the same exercise at normoxia.

However, the response of the corticospinal pathway at hypoxia has been mostly investigated at rest or after exercise, but using exercise protocols related to endurance-like activities, such as cycling (Goodall et al. 2012; Amann et al. 2013) or long-lasting submaximal contractions (Millet et al. 2009, 2012; Ruggiero et al. 2018). At rest, there is some inconsistency about the effect of acute hypoxia on CSE. Some studies found increases in CSE [measured as the amplitude or the area of the motor evoked potential (MEP)] during exposure to different hypoxic protocols, while others did not show changes in response to acute hypoxic exposure (Goodall et al. 2010; Millet et al. 2012). This inconsistency may be related to the hypoxia severity and/or duration (Rupp et al. 2012). Indeed, most of the available research has been conducted under moderate to severe acute hypoxia (FiO_2_ between 0.15 and 0.10) (Millet et al. 2009, 2012; Goodall et al. 2012; Amann et al. 2013; Ruggiero et al. 2018). However, most of training camps used by athletes are developed at training facilities between 1800 and 2500 m asl, which is associated with lower levels of hypoxia (low to moderate, FiO_2_ between 0.18 and 0.15) (Dietz and Hackett 2019). Therefore, from a practical point of view, seems logical to test the effects of hypoxic levels usually associated with the altitude where training camps occur.

Therefore, the present study aimed to test whether an acute ascending to a moderate altitude (2320 m asl), affects CSE at rest, before, during and after a traditional high-volume hypertrophy-oriented *R*_T_ session, as expressed by changes in the MEP amplitude evoked by means of TMS. Furthermore, we also aimed to elucidate whether a possible alteration in the CSE could be related to changes in the excitability of the intracortical circuitry using a paired-pulse protocol [short-interval intracortical inhibition (SICI)]. We also aim to explore if changes in the excitability of the brain to muscle structures (SICI, CSE and M-wave) are associated with differences in metabolic [blood lactate concentration (BLa)], perceptual [ratings of perceived exertion (RPE) and perceived muscular pain] and performance variables recorded through the *R*_T_ session. We hypothesized that acute exposure to a moderate altitude would increase CSE due to a lower inhibition of the cortico-cortical structures (SICI) and lead to a maintenance of the performance during the *R*_T_ session in spite of a higher metabolic stress and effort perception caused by hypoxia with respect to the same *R*_T_ session in normoxia.

## Materials and methods

### Participants

Twelve resistance-trained men (self-reported consistent participation in *R*_T_ program with a training frequency ≥ 2 sessions per week for at least 12 months before the experiment) volunteered to participate in this study (age: 22.3 ± 3.1 years; height: 174.9 ± 4.7 cm; body mass: 72.9 ± 8.9 kg; all right handed). Participants had no self-reported health, neurological or muscular disorders and were not exposed to more than 3 consecutive days of altitudes above 1500 m asl for at least 3 months before the study. All participants were informed in detail about the experimental protocol before signing the informed consent and were instructed to abstain from physical activity and alcohol intake and to maintain their daily sleep and diet habits for 72 h before any evaluation. This study was approved by the Andalusian Government Research Ethics Committee (Ethical Application Ref. CEIM/CEI 10/18) and conducted in accordance with the Helsinki Declaration.

### Study design

Each participant completed a preliminary assessment session under normoxic conditions and two experimental trials during which participants completed a traditional hypertrophy *R*_T_ session at moderate terrestrial altitude (H; at the High Performance Center of Sierra Nevada, located at 2320 m asl) or normoxia (N; at the Sport Science Faculty of University of Granada, which is located at < 700 m asl). Each training session consisted of 8 sets of 10 repetitions at 70% of one repetition maximum (1RM) of a biceps curl (70% of 1RM = 21.4 ± 7.6) and 90 s of inter-set rest. The hypoxic environmental condition was evaluated before the *R*_T_ session by assessing the arterial oxygen saturation (SpO_2)_ using a pulsioximeter (Wristox 3100; Nonin, Plymouth, MN, USA). Mean rest SpO_2_ values for N and H conditions were 98.0 ± 0.9% and 94.0 ± 1.9%, respectively. The two experimental trials were randomized and separated by 48 h of rest and performed at the same time of day under the following laboratory conditions: ∼ 22 °C and ∼ 60% humidity, or ∼ 22 °C and ∼ 28% humidity, respectively for N and H.

Body mass (Tanita BC 418 segmental, Tokyo, Japan) and height (Seca 202, Seca Ltd., Hamburg, Germany) were measured during the preliminary session. In a preliminary session, after a standard warming-up, subject performed 3 sets of 4–6 repetitions at submaximal load (perceived 50–60–75% of 1RM) of biceps curl on an EZ-Scott bench. The exercise was performed in a supination position and full range of movement. Then, they rested for 5 min. After that, subject completed 1 set of 4–6 repetitions to failure. Using the 4–6RM load, 1RM values were predicted using Brzycki’s equation (Brzycky 1993), validated for biceps curl (Hutchins and Gearhart 2010).

**Fig. 1:**
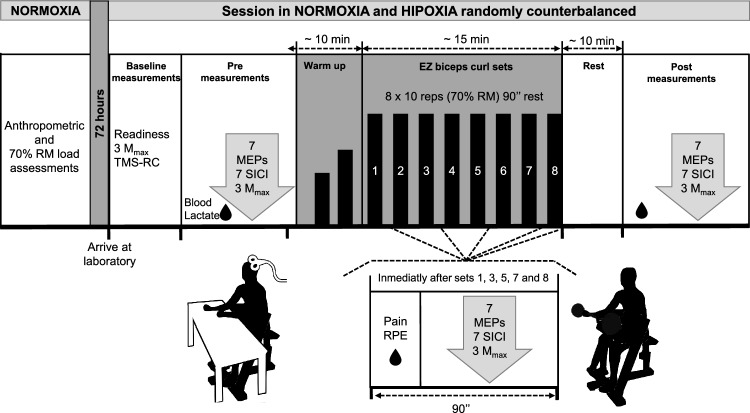
Schematic view of the set-up and protocol

During the experimental trials, before each *R*_T_ session (10 min after arrival at the laboratory), the subjective well-being of participants and neuromuscular measurements were assessed to account for the effect of acute hypoxic exposure at rest (Fig. 1). For the neuromuscular assessment, electrical stimulation of the brachial plexus (maximal compound muscle action potentials [*M*_max_]) and a single-pulse TMS of the biceps brachii (BB) were performed to assess neuromuscular transmission and CSE, respectively. Baseline CSE evaluation consisted of a recruitment curve measured at rest, with four pulses at each stimulation intensity, starting with a subthreshold intensity of 30% of the stimulator output and increasing stimulation intensity in steps of 10% until 90% of the maximal stimulator output.

Immediately before the start of *R*_T_ sessions (Pre), after the 1st, 3rd, 5th, 7th and 8th set and 10 min after exercise cessation (Post), blood lactate concentration [BLa; Lactate Pro 2 device (Arkray, Japan)], perceptual (RPE-10 and muscle pain) and neuromuscular responses were measured. Each block of neuromuscular measurements consisted of three *M*_max_, seven single-pulse MEPs and seven paired-pulse MEPs to test for acute changes in neuromuscular transmission, CSE and SICI, respectively (Fig. 1).

### Procedures

#### Set up

During testing, participants sat on a commercially standard Scott bench (Techogym, Inc) with their trunk in a vertical position (90°). Their axillae and arms were supinated and resting on the frontal pad (45° of shoulder flexion) and the forearms rested horizontally on a foam pad located on a table (see Fig. 1). The bench height was adjusted for each participant, so the trunk remained straight and both feet firmly on the floor. After skin preparations (shaving, abrading and cleaning with alcohol), Ag–AgCl surface electromyography (EMG) electrodes were positioned over the right BB muscle belly (2 cm interelectrode distance) following SENIAM recommendations (Hermens et al. 2000). EMG was amplified (× 200), band-pass-filtered (10–1000 Hz), and sampled (2 kHz) with a Digitimer d440 isolated amplifier (Digitimer Ltd, Welwyn Garden City, Uk) connected to data acquisition interface and software (CED Micro1401-3, Signal v6, Cambridge Electronic Design Ltd, Cambridge, UK).

#### Subjective well being scale

When arriving to the laboratory, in each experimental session (i.e.: H and N), a well-being questionnaire was fulfilled. Participants recorded subjective ratings of sleep quality (1 = Insomnia, 5 = Okay, 9 = Very Restful), fatigue (1 = Always Tired, 5 = Okay, 9 = Very Fresh), muscle soreness (1 = Very Sore, 5 = Okay, 9 = Feeling Great), stress (1 = Very Stressed, 5 = Okay, 9 = Very Relaxed) and mood (1 = Irritable, 5 = Okay, 9 = Very Positive) on a 9-point numerical scale (Hooper et al. 1995).

#### Perceptual responses

RPE-10 was obtained via a Category Ratio-10 scale viewed by participants immediately after the 1, 3, 5, 7 and 8 set (Foster et al. 2001). The participants were also asked to scale their pain in the BB using the Borg CR-10 scale. The instructions used to guide participants during the perceptual assessment were the same as those recommended by Borg (1998).

#### Brachial plexus stimulation

For recording the *M*_max_ in the BB, single-pulse stimulation (200 µs duration) was delivered to the brachial plexus with a DS7AH constant current electrical stimulator (Digitimer Ltd, Welwyn Garden City, Uk). The cathode (pre-gelled Ag–AgCl electrodes) was positioned in the supraclavicular fossa and the anode on the acromion. After defining the stimulation intensity needed to evoke the *M*_max_ in the BB, the intensity was set to 130% of this value for the measurements (98 ± 46 mA). The peak-to-peak amplitude of *M*_max_ was measured offline, averaged for each block and used for normalization of all other EMG variables to control for membrane excitability and propagation changes (Rodriguez-Falces and Place 2017).

#### Transcranial magnetic stimulation (TMS)

Single- and paired-pulse TMS was delivered to the left motor cortex (M1) with a figure of eight coil (70 mm diameter) connected to two DuoMAG (Rogue Resolutions Ltd.) magnetic stimulators. The coil was oriented with the handle at ~ 45° posterolaterally to the midline, and the optimal stimulation location was obtained by exploring the estimated center of the BB motor cortical representation. The point where stimulation produced the largest MEP in the right BB was marked directly on the scalp with a permanent marker. Resting motor threshold (rMT) was defined as the lowest stimulation intensity needed to obtain three out of five MEPs of a peak-to-peak amplitude > 50 µV. To measure SICI, a paired-pulse protocol was used in which the intensity of the conditioning stimulus (CS) was set at 80% of the rMT, while the intensity for the test stimulus (TS), delivered 2.5 ms later, was set at 140% of the rMT. The peak-to-peak amplitude of MEPs was measured offline. The amplitude of the MEPs obtained at baseline during the recruitment curve was used to calculate the total area under the recruitment curve (AURC) using the trapezoidal integration method. CSE immediately before, during and after the *R*_T_ sessions was computed by averaging the peak-to-peak amplitude of the seven unconditioned single pulse MEPs of each block. SICI was quantified by dividing the average of the seven paired-pulse MEPs by the averaged single-pulse MEPs of each block and multiplying by 100. The root mean square of the EMG (rmsEMG) during the 150 ms previous to every pulse was also measured and averaged for each session to ensure identical background EMG conditions.

### Resistance training session

Before each *R*_T_ session, participants undertook a standard warm-up protocol consisting of 10 min of row machine aerobic exercise and stretching exercises followed by a specific warm-up comprised by 2 sets of 10 biceps curl repetitions (the first with 6 kg and the second at 50% 1RM estimated from the preliminary session, 90 s rest) in the EZ-Scott bench.

Each *R*_T_ session consisted of 8 sets of 10 biceps curl repetitions on the EZ-Scott bench with a load of 70% of 1RM and 90 s of inter-set rest. The training session was performed on the same bench and position in which the neuromuscular evaluation was made. The cadence of repetitions was carried out in a controlled fashion, with a concentric contraction of approximately 1 s and an eccentric contraction of roughly 2 s as determined by the supervising researcher. The load was reduced a 10% in those cases in that participants reached volitional failure before achieving the target repetition range (8–10 repetitions) with respect to the previous set. Absolute training load (kg) and repetitions to failure were monitored during each training session. Total volume was calculated as the sum of the load lifted multiplied by the repetitions of each set (Scott et al. 2016a).

### Statistical analyses

Normality was tested using the Saphiro Wilk’s test. To determine the effects of hypoxia at rest, a paired t test analysis or, if data was not normally distributed, a Wilcoxon signed-rank test were performed for well-being (sleep, pain, fatigue and stress) and neuromuscular values (rMT, AURC, MEP_max_ and *M*_max_). Another t test was performed to determine possible differences in training variables between normoxic and hypoxic conditions. A two-way repeated measures analysis of variance (RM-ANOVA) was performed with time (Pre, set 1, set 3, set 5, set 7, set 8 and Post) and condition (N and H) as factors for the following variables: *M*_max_, MEP/*M*_max_, SICI, BLa. If sphericity was violated (Mauchly's test), degrees of freedom were corrected by Greenhouse–Geisser estimates of sphericity. When significant interactions were found in the RM-ANOVA, planned comparisons (Pre vs. Set 1; Pre vs. Set 3; Pre vs. Set 5; Pre vs. Set 7; Pre vs. Set 8 and Pre vs. Post) were made with Bonferroni correction [*P* value × 6 (i.e., number of planned comparisons)] to account for multiple comparisons. For RPE and Pain and any other variable not normally distributed even after log transformation, we used a non-parametric ANOVA-type test (Noguchi et al. 2012). When significant main effects or interactions were found with the non-parametric ANOVA-type test, planned comparisons (RPE and Pain: Set 1 vs. Set 3; Set 1 vs. Set 5; Set 1 vs. Set 7; Set 1 vs. Set 8) were made with a Wilcoxon signed rank test with Bonferroni correction (*P* value × 4 (i.e., number of planned comparisons) to account for multiple comparisons. ES are presented as partial eta-squared values (*η*_p_^2^; small: 0.01; medium: 0.06; large: 0.14) for the factors of the RM-ANOVAs and as Cohen's d for the paired comparisons. Threshold classifications were set as follows: > 0.2 [small], > 0.6 [moderate], > 1.2 [large] and > 2 [very large] (Hopkins et al. 2009). Unless indicated otherwise, data are reported as means and SD. SPSS 20.0 software (SPSS, Chicago, IL) was used for statistical analysis. Statistical significance was set at *P* ≤ 0.05.

## Results

### Baseline measures on different environmental conditions

Subjective ratings of sleep quality (*P* = 0.92; *Z* = − 0.11, ES = − 0.09), fatigue (*P* = 0.96; *Z* = − 0.05, ES = − 0.06), muscle soreness (*P* = 0.22; *Z* = − 1.22, ES = − 0.39) and stress (*P* = 0.68; *Z* = − 0.41 ES = − 0.09), were not different between N and H (see Table 1).

**Table 1:** Descriptive data of the well-being and neuromuscular baseline data obtained before each resistance training session performed under normoxic and hypoxic conditions

	Normoxia	Hypoxia
Wellbeing		
Sleep (a.u.)	7.6 ± 1.1	7.7 ± 1.2
Pain (a.u.)	1.9 ± 1.3	2.7 ± 2.3
Fatigue (a.u.)	2.3 ± 1.2	2.4 ± 1.9
Stress (a.u.)	1.8 ± 0.6	1.8 ± 1.1
Neuromuscular		
rMT (%MSO)	46.8 ± 6.6	44.3 ± 6.5^a^
AURC (a.u.)	153.2 ± 76.9	193.2 ± 145.4
MEP_max_ (%*M*_max_)	6.4 ± 2.7	8.3 ± 5.4
*M*_max_ (mV)	9.0 ± 2.2	8.7 ± 2.2

a.u., arbitrary units; rMT, resting motor threshold; %MSO, percentage of the maximum stimulator output; AURC, area under the recruitment curve; MEP, motor evoked potential; *M*_max_, maximal compound muscle action potential

^a^*P* < 0.05

**Fig. 2:**
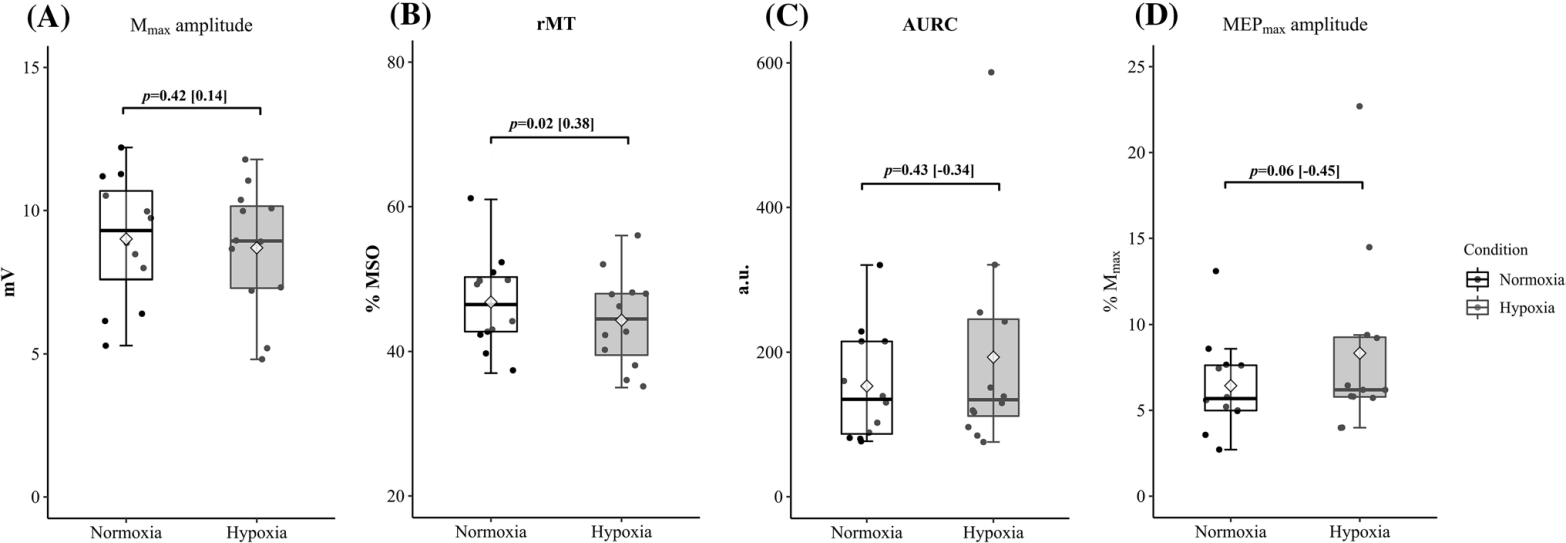
Mean and individual values for *M*_max_ (**A**), rMT  (**B**), AURC  (**C**) and MEP_max_ (**D**) obtained before each resistance training session performed under normoxic (left column) and hypoxic (right column) conditions

The rMT was a 5.3% lower in H than in N (*P* = 0.02; ES = 0.38). No differences were found in AURC (*P* = 0.43; *Z* = − 0.78, ES = − 0.34), MEPmax (*P* = 0.06; *Z* = − 1.88, ES = − 0.45) or *M*_max_ (*P* = 0.42; ES = 0.14) between N and H (see Table 1 and Fig. 2).

### *R*_T_ session acute effects on different environmental conditions

Total training volume was not different between N (1618 ± 468 kg) and H (1638 ± 509 kg; *P* = 0.51; ES = − 0.04). Mean RPE-10 was a 12% greater during the *R*_T_ session at H than at N (*P* < 0.001; *Z* = − 4.57, ES = − 0.53; see Table 2 for raw values, main effects and interactions). Regardless the environmental condition (time main effect), RPE-10 after sets 3, 5, 7 and 8 was greater than RPE-10 after set 1 (all *P* < 0.01, *Z* = 3.78–4.24, ES = 0.73–1.81; see Table 2). Similarly, BB pain was a 54% greater during the *R*_T_ session at H than at N (*P* < 0.001; *Z* = − 5.74, ES = − 1.34; see Table 2 for raw values, main effects and interactions). Regardless of environmental condition, BB pain was greater after sets 3, 5, 7, and 8 compared to the first set (all *P* < 0.01, *Z* = 3.62–3.95, ES = 0.50–1.30; see Table 2).

**Table 2:** Mean values (± SD) and statistics results (two-way repeated measures parametric analysis of variance (ANOVA) or non-parametric ANOVA-type test) for neuromuscular, metabolic and perceptual responses during and after resistance training sessions performed under normoxic and hypoxic conditions

Variable	Condition	Time							Statistics		
		Pre	Set_1	Set_3	Set_5	Set_7	Set_8	Rec_10′	Condition	Time	Cond × Time
RPE (a.u.)	N	–	3.5 ± 1.4	4.5 ± 1.7	5.9 ± 1.5	6.5 ± 1.8	6.6 ± 1.8	**–**	*F*_1, ∞_ = 16.37, *P* < 0.001	*F*_1,86, ∞_ = 25.30, *P* < 0.001	*F*_*2*,28, ∞_ = 0.49, *P* = 0.63
	H	–	4.3 ± 1.7	5.3 ± 1.8	6.3 ± 1.6	7.3 ± 1.7	7.3 ± 1.8	**–**			
Pain (a.u.)	N	–	1.6 ± 1.2	2.5 ± 1.5	3.7 ± 1.9	4.0 ± 2.4	4.5 ± 2.6	**–**	*F*_1, ∞_ = 16.32, *P* < 0.001	*F*_1.45, ∞_ = 19.47, *P* < 0.001	*F*_1.96, ∞_ = 1.96, *P* = 0.22
	H	–	3.3 ± 1.9	4.3 ± 2.0	4.9 ± 2.2	6.2 ± 2.4	6.4 ± 2.5	**–**			
BLa (mMol·l^−1^)	N	1.4 ± 0.4	4.0 ± 1.5	4.8 ± 1.5	5.3 ± 1.6	5.5 ± 1.5	5.4 ± 1.7	4.7 ± 1.1	*F* (1, 11) = 9.44; *P* = 0.01; *η*_p_^2^ = 0.46	*F* (3, 33.1) = 39.11; *P* = 0.001; *η*_p_^2^ = 0.78	*F* (6, 66) = 3.30; *P* = 0.007; *η*_p_^2^ = 0.23
	H	1.4 ± 0.3	4.7 ± 1.9	5.6 ± 2.2	6.2 ± 1.8	6.8 ± 2.2	6.2 ± 2.3	4.7 ± 1.4			
*M*_max_ (mV)	N	8.5 ± 2.4	9.4 ± 1.7	9.6 ± 1.7	9.4 ± 1.9	9.4 ± 2.1	9.2 ± 2.2	8.4 ± 2.5	*F*_1, ∞_ = 0.56, *P* = 0.45	*F*_*2*.72, ∞_ = 8.67, *P* < 0.001	*F*_*3*.38, ∞_ = 0.55, *P* = 0.67
	H	8.6 ± 2.2	9.1 ± 2.2	9.2 ± 2.2	9.2 ± 2.2	9.2 ± 2.2	8.8 ± 2.2	8.0 ± 2.2			
MEP (%*M*_max_)	N	4.9 ± 2.5	4.0 ± 2.9	3.7 ± 2.7	3.5 ± 2.8	3.8 ± 3.1	4.0 ± 2.9	4.4 ± 2.6	*F* (1, 11) = 0.18; *P* = 0.68; *η*_p_^2^ = 0.02	*F* (6, 66) = 6.99; *P* = 0.001; *η*_p_^2^ = 0.39	*F* (3.2, 35.7) = 0.86; *P* = 0.53; *η*_p_^2^ = 0.07
	H	4.5 ± 3.0	3.4 ± 2.8	3.1 ± 2.3	3.3 ± 1.9	3.4 ± 2.7	3.6 ± 2.5	4.8 ± 3.8			
SICI (%)	N	49.3 ± 20.0	60.1 ± 20.0	59.5 ± 23.2	66.3 ± 26.1	60.4 ± 15.3	58.4 ± 22.9	62.4 ± 22.7	*F* (1, 11) = 0.02; *P* = 0.90; *η*_p_^2^ = 0.01	*F* (6, 66) = 1.55; *P* = 0.18; *η*_p_^2^ = 0.12	*F* (6, 66) = 1.17; *P* = 0.33; *η*_p_^2^ = 0.10
	H	54.7 ± 14.2	64.6 ± 20.3	61.9 ± 24.6	55.3 ± 17.8	59.0 ± 14.2	64.4 ± 20.9	59.5 ± 13.2			

*Mmax* maximal compound muscle action potential, *MEP* motor evoked potential, *SICI* short-interval intracortical inhibition, *La* blood lactate concentration, *RPE*: rate of perceived exertion, *a.u.* arbitrary units

**Fig. 3:**
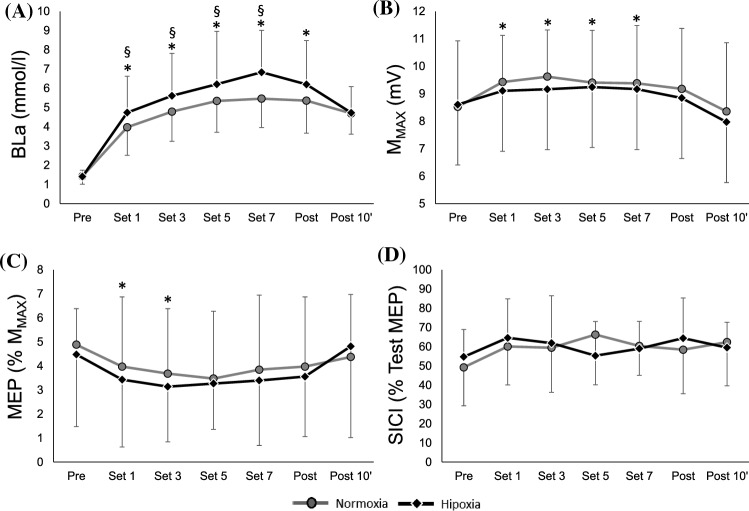
Mean values for BLa (**A**), *M*_max_ (**B**), MEP (**C**) and SICI (**D**) values after set 1, 3, 5, 7, 8 (post) and 10 min after the last set (post 10′) of the *R*_T_ session performed at N and H. * Shows statistically significant (*P* < 0.05) differences with Pre; § shows statistically significant (*P* < 0.05) differences between environmental conditions

BLa was significantly greater after every set compared to Pre values at N (all *P* < 0.001; ES = 2.37–3.66) and H (all *P* < 0.001; ES = 2.44–3.81). BLa was greater at H than N after set 1 (*P* = 0.001; ES = − 0.44), 3 (*P* = 0.05; ES = − 0.43), 5 (*P* = 0.02; ES = − 0.51), and 7 (*P* = 0.004; ES = − 0.73) but not after 8 (*P* = 0.11; ES = − 0.42) or at Post (*P* = 0.92; ES = − 0.03, see Fig. 3). Similarly, regardless of environmental conditions, *M*_max_ was significantly greater after set 1, 3, 5 and 7 compared to Pre values (all *P* < 0.05; *Z* = 3.04–3.32, ES = 0.18–0.38), but it recovered after 10 min of rest (Post; *P* = 0.25; *Z* = 2.04, ES = 0.18). MEP was slightly reduced compared to Pre but reduction was only significant after the first (− 21%, *P* = 0.036; ES = 0.53) and third set (− 27%, *P* = 0.024; ES = 0.57) and it was fully recovered ten minutes after finishing the *R*_T_ sessions (− 2% *P* = 0.99; ES = 0.15; see Fig. 3) regardless of environmental conditions. SICI did not change after an *R*_T_ session in any environmental condition.

## Discussion

We determined the effects of acute of hypobaric hypoxia and its interaction with a dynamic high-volume hypertrophy-oriented *R*_T_ session of the BB on neuromuscular and perceptual responses. Acute exposure to hypobaric hypoxia reduced the rMT despite no changes in the corticospinal input–output relationship, subjective well-being, or neuromuscular transmission. At H, a single session of *R*_T_ of the BB led to a greater BLa, RPE and BB pain than N. However, CSE decreased slightly during the *R*_T_ session without changes in intracortical inhibition, regardless of the environmental condition. The data suggest that despite the effects of acute hypobaric hypoxia exposure on rMT and the greater subjective effort levels and BLa concentration during a single *R*_T_ session performed at H, corticospinal responses were similar to those experienced during a *R*_T_ session at normoxia.

### Baseline measures on different environmental conditions

Acute exposure to hypoxia (either hypobaric or normobaric hypoxia) leads to significant reductions in SpO_2_ at rest compared to normoxia. Our results show that the 2300 m of terrestrial altitude were sufficient to induce an immediate state of moderate hypoxia in participants (− 4% SpO_2_), a reduction similar to that experienced in previous studies in which participants breathed hypoxic gas mixtures (normobaric hypoxia, fraction of inspired oxygen (FiO_2_ = 0.16) (Goodall et al. 2010).

This moderate hypoxia was accompanied by a significant reduction in rMT but without significant changes in MEP amplitudes or SICI, suggesting only a mild increase in CSE. Our results are in line with those from Szubski et al. (2006) in which reductions in rMT, but no changes in MEP amplitude or intracortical inhibition, were present after acute hypoxia (FiO_2_ = 0.12), which may reflect changes in the most excitable neurons of the corticospinal pathway, the ones that determine the motor threshold (Devanne et al. 1997; Carroll et al. 2001). Although we cannot discard changes at the spinal level (Štirn et al. 2022), results regarding the effects of acute sustained hypoxia are inconsistent (Szubski et al. 2006; Rupp et al. 2012). Therefore, the reduction in rMT may be related to changes at the cortical level. Specifically, the lack of changes in intracortical inhibition measured by paired-pulse TMS suggests that cortical changes may be related to membrane hyperexcitability of the cortical neurons, rather than to changes in synaptic GABAergic inhibitory mechanisms (Ziemann et al. 1996; Chen et al. 1997; Boroojerdi et al. 2001). Nevertheless, although our results confirm those from previous studies, we must be cautious because of the slight change in rMT, which was not accompanied by increases in MEP amplitude. This subtle to no changes in CSE and intracortical inhibition, respectively, could be related to the mild hypoxic effect induced by the 2300 m of terrestrial altitude, or the time dependent effects of acute hypoxia at the corticospinal level. Indeed, Rupp et al. (2012) found significant increases in CSE only after 3 h but not 1 h of hypoxic breathing (FiO_2_ = 0.12). Therefore, despite the fact that the ~ 45 min of hypoxia exposure before baseline measurements were sufficient to induce a significant reduction in SpO_2_, this time exposure may have been too short to cause significant changes in the corticospinal structures mediating the responses to single- and paired-pulse TMS.

### R_T_ session acute effects on different environmental conditions

We hypothesized that the larger anaerobic metabolism contribution during *R*_T_ at H and its potential influence over motor unit recruitment would increase the corticospinal demands of a single *R*_T_ session, leading to greater acute increases in corticospinal and/or intracortical excitability.

The decreased oxygen availability associated with H increases anaerobic glycolysis contribution to ATP synthesis, leading to a larger metabolite build-up during *R*_T_ at hypoxia (Balsom et al. 1994; Kon et al. 2010; Feriche et al. 2017). Indeed, although training volume performed during *R*_T_ training was similar at H and N, we found that the 2300 m of terrestrial altitude reduced SpO_2_ and increased blood lactate accumulation during the *R*_T_ session, suggesting a larger anaerobic metabolism contribution to energy supply during exercise. This more significant metabolic stress could lead to increased group III and IV afferents discharge with an inhibitory effect on α motoneuron pool (Rotto and Kaufman 1988; Scott et al. 2014). To compensate the inhibitory effect over firstly recruited lower threshold motor units, an increase in supraspinal drive may be needed to recruit higher threshold motor units and/or increase the firing rate of the already recruited motor units to produce the muscle force required to lift the external load (Scott et al. 2014; Feriche et al. 2017). This greater supraspinal drive may suppose a greater demand during a *R*_T_ session, leading to greater acute effects on cortical and corticospinal structures at H. Indeed, previous studies have found greater increases in CSE and/or intracortical inhibition when using higher *R*_T_ intensities that require a greater supraspinal drive (Colomer-Poveda et al. 2019, 2020; Mason et al. 2019), which suggest that supraspinal drive during *R*_T_ could have a dose–effect over acute corticospinal changes after a *R*_T_ session.

However, in contrast to our initial hypothesis, despite the perceptual and metabolic differences during the high-volume hypertrophy-oriented *R*_T_ session performed at N and H, both sessions had similar effects on the corticospinal pathway and neuromuscular transmission. In the present study, we showed that a high-volume hypertrophy-oriented *R*_T_ session had no effect on intracortical inhibition but acutely decreased CSE despite the acute increase in neuromuscular transmission. The lack of differences between H and N could be related to the high effort associated with the high-volume hypertrophy-oriented *R*_T_ session. The high intensity used and the sets performed close to or reaching concentric muscle failure, may have reduced potential differences in the corticospinal drive between H and N despite the greater anaerobic involvement during the *R*_T_ session at H. Additionally, the moderate hypobaric hypoxia induced by a short time (30–60 min) exposure to 2300 m of terrestrial altitude may have smoothed the H effects, as suggests the slight change in the rMT at rest. Recent studies propose that intermittent severe hypoxia could have a greater effect on corticospinal plasticity, even at rest (Christiansen et al. 2018). Therefore, not only duration and intensity, but also the pattern of hypoxia exposure may be important to induce corticospinal plasticity. Future studies should test if acute intermittent hypoxia-induced corticospinal plasticity may optimise *R*_T_-induced spinal and/or supraspinal plasticity.

The acute reduction in CSE during the *R*_T_ session found in the present study contrasts with some previous findings. However, the results regarding the acute effects of a *R*_T_ session over the corticospinal tract are inconsistent, with studies showing increases (Selvanayagam et al. 2011; Nuzzo et al. 2016; Latella et al. 2018), decreases (Latella et al. 2016) or no changes (Jensen et al. 2005; Ruotsalainen et al. 2014; Brandner et al. 2015) in CSE and SICI after a *R*_T_ session. These acute changes have been suggested to be related to a strengthen of the cortical and corticospinal connections to the muscles involved in the task due to use-dependent plasticity, which may be linked to the learning process implicated in the early performance improvements that occur with *R*_T_ (Nuzzo et al. 2016; Colomer-Poveda et al. 2019; Mason et al. 2020). However, given the inconsistencies about the effects of a single session of *R*_T_ over the cortical and corticospinal tract, caution should be taken when interpreting those acute changes as the initial neural adaptations with functional implications over the long-term (Colomer‐Poveda et al. 2021; Hortobágyi et al. 2021). Indeed, no study up to date, have shown any relations between acute corticospinal and/or intracortical changes after a *R*_T_ session with long-term functional or neural adaptations. Furthermore, as occurred in our study, there are also other research showing no changes, or even reductions in CSE immediately after a single dynamic heavy load-*R*_T_ protocol compatible with the ones recommended by *R*_T_ guidelines that will potentially increase muscle force when repeated in time (Latella et al. 2016).

Alternatively, those acute changes may represent a consequence of the fatigue imposed by the *R*_T_ session, reducing motoneuron and/or cortical excitability (Todd et al. 2003). For example, reductions in MEPs have been reported after sustained submaximal isometric contractions of the elbow flexors (Todd et al. 2003; McNeil et al. 2011). Therefore, acute corticospinal changes could reflect the demands of the task over the corticospinal tract structures that may trigger long-term adaptations despite not having the same magnitude and direction (decrease vs. increase) of the presumed chronic neural adaptations occurring after repeated *R*_T_ sessions. Indeed, we cannot discard a delayed enhancement in CSE as occurred after a similar BB dynamic heavy *R*_T_ session, where MEP was immediately decreased after the *R*_T_ session but increased compared to baseline after 72 h of rest (Latella et al. 2016).

In conclusion, the data tentatively suggest that acute exposure to 2300 m of moderate terrestrial altitude slightly increased the excitability of the most excitable structures of the corticospinal tract. However, a single dynamic high-volume hypertrophy-oriented *R*_T_ session transiently reduced CSE and increased neuromuscular transmission without effects on intracortical inhibition, regardless of the environmental condition. These results suggest that moderate hypobaric hypoxia does not influence corticospinal and intracortical responses to a single hypertrophy-oriented *R*_T_ session of the BB. However, this may be partially explained by the relatively small metabolic stress generated by the exercise used in the present study (i.e.: biceps curl), and the low muscle mass involved (i.e.: BB). Therefore, further studies should test the effects on the neuromuscular function of other type of *R*_T_ exercises involving greater amounts of muscle mass (e.g.: back squat), and thus higher metabolic stress and III and IV afferent signaling.

## Data Availability

The datasets generated during and/or analysed during the current study are available from the corresponding author on reasonable request.

## Abbreviations

asl: Above sea level
AURC: Area under the recruitment curve
BB: Biceps brachii
BLa: Blood lactate concentration
CSE: Corticospinal excitability
EMG: Surface electromyography
FiO_2_: Fraction of inspired oxygen
H: Hypoxic environmental condition
MEP: Motor evoked potential
*M*_max_: Maximal compound muscle action potential
N: Normoxic environmental condition
RM-ANOVA: Repeated measures analysis of variance
rmsEMG: Root mean square of the surface electromyography
rMT: Resting motor threshold
RPE: Rate of perceived exertion
*R*_T_: Resistance training
SICI: Short interval intracortical inhibition
SpO_2_: Arterial oxygen saturation
TMS: Transcranial magnetic stimulation
1RM: One repetition maximum
